# Imaging cortical activity following affective stimulation with a high temporal and spatial resolution

**DOI:** 10.1186/1471-2202-10-83

**Published:** 2009-07-17

**Authors:** Julian Keil, Hannah Adenauer, Claudia Catani, Frank Neuner

**Affiliations:** 1 Department of Psychology, University of Konstanz and Center for Psychiatry Reichenau, D-78457 Konstanz, Germany; 2 Department of Psychology, University of Bielefeld, D-33501 Bielefeld, Germany

## Abstract

**Background:**

The affective and motivational relevance of a stimulus has a distinct impact on cortical processing, particularly in sensory areas. However, the spatial and temporal dynamics of this affective modulation of brain activities remains unclear. The purpose of the present study was the development of a paradigm to investigate the affective modulation of cortical networks with a high temporal and spatial resolution. We assessed cortical activity with MEG using a visual steady-state paradigm with affective pictures. A combination of a complex demodulation procedure with a minimum norm estimation was applied to assess the temporal variation of the topography of cortical activity.

**Results:**

Statistical permutation analyses of the results of the complex demodulation procedure revealed increased steady-state visual evoked field amplitudes over occipital areas following presentation of affective pictures compared to neutral pictures. This differentiation shifted in the time course from occipital regions to parietal and temporal regions.

**Conclusion:**

It can be shown that stimulation with affective pictures leads to an enhanced activity in occipital region as compared to neutral pictures. However, the focus of differentiation is not stable over time but shifts into temporal and parietal regions within four seconds of stimulation. Thus, it can be crucial to carefully choose regions of interests and time intervals when analyzing the affective modulation of cortical activity.

## Background

Research in affective neuroscience supports the notion that emotional cues guide selective visual attention and receive enhanced processing [1-4]. In a study using functional magnetic resonance imaging (fMRI), Lang and collaborators [5] found that the functional activation in visual areas of the occipital cortex varied as a function of affective arousal. Other fMRI studies revealed increased BOLD (Blood Oxygen Level Dependent) signals in associative visual regions and subcortical limbic structures when viewing emotionally arousing compared to neutral pictures [6,7]. However, as fMRI measures of blood oxygen level dependent responses take several seconds to build up, they are not able to provide information about temporal characteristics of emotional picture processing. Given the low temporal resolution of the BOLD response and its relation to metabolic processes rather than to neuronal functioning, it has been suggested to use measures that complement the information obtained by hemodynamic imaging techniques [8] and thus provide additional information on the temporal characteristics of emotional picture processing [9]. While the combination of the measurement of the BOLD-signal in the fMRI and visual evoked potentials (VEP) promises to solve the problem of the low temporal resolution, this combination might not lead to more accurate results, as two different processes could be active, namely the fast transients of the event related potential and the slow change of blood flow.

In accordance with this notion, an early difference in the visual processing of emotional (pleasant and unpleasant) compared to neutral pictures is revealed by the early posterior negativity (EPN) developing around 120–150 ms after stimulus onset and lasting until about 300 ms [10,11]. This temporo-occipital cortical ERP component reflects the selective processing of emotional stimuli. The amplitude of this component is most pronounced for stimuli of high evolutionary significance. Hence, the early differential ERP response may reflect a very early processing advantage of affective stimuli at the initial stages of perceptual processing [12]. These findings suggest that the visual cortex is differentially activated as a function of emotional arousal. In addition to these findings in very early time domains, ERP studies have consistently demonstrated a sustained late positive potential (LPP) (past 300 ms) in response to emotional stimuli compared to neutral ones [13]. This posterior cortical ERP component reflects the recruitment of selective attentional processes with respect to motivational significant stimuli. Keil et al. [14] showed a differentiating response to emotionally arousing and neutral pictures in late event related components.

In addition to temporal changes in emotional processing, these studies provide information about the spatial dynamics of affective picture processing. Keil [14] found that the emotional processing within the first milliseconds after stimulus onset is not limited to the primary visual cortex. This study also revealed that, with increasing viewing time (up to 900 ms post stimulus), affect modulation extended from inferior-posterior to higher order visual cortical areas such as parietal and occipito-temporal regions. These spatial changes can be seen as correlates of longer lasting, higher order processing structures.

However, little is known about the time course of activation in response to emotional stimuli after these early and late event related potentials. Also, as some ERP analyses calculate an average over large sensor clusters, the spatial resolution of the aforementioned studies could significantly be improved by examining the source of activation on the dipole level. Looking at source estimations of activation rather than the recorded topography allows identifying the location of the activation of interest with greater accuracy. While analyzing the topography of activation can be very useful in identifying the general components of the ERP, this procedure lacks the ability to distinctly distinguish between cortical regions. Thus, there is a lack of a reliable and accurate analytical framework to comprise both spatial and temporal changes with high accuracy. Using a source-estimation technique on high-resolution time course data unifies both approaches of an accurate identification of cortical activation and detailed examination of modulations of activation over time. In turn, this would allow the analysis of the network associated with processing of emotional information step-by-step over a long time interval. This might shed light onto the temporal sequence of cortical activation involved in this process. This is especially important given recent findings in our own group that the processing of emotional content is disturbed in anxiety disorders, most prominently Posttraumatic Stress Disorder PTSD [15].

The present study examines an approach to analyze the time course of physiological data that is both spatially and temporally accurate as well as easy and fast to compute. One possibility to account for temporal changes is the steady-state design. The steady-state visual evoked potentials (ssVEPs) (or the steady-state visual evoked field, ssVEF in case of neuromagnetic data) represent a continuous brain response elicited by a repetitive visual stimulus presented at a certain frequency (e.g. 10 Hz). As ongoing cortical oscillatory responses, they have the same fundamental frequency as the driving stimulus [16]. One major advantage of the ssVEF technique is that the response of interest can be examined at high temporal resolution and signal-to-noise ratio even when the number of trials is limited. Furthermore, ssVEF data can also be used to investigate the time course of activation over longer time periods up to some seconds [17]. Studies using this paradigm have shown that high arousing pictures generate greater ssVEFs or ssVEPs than neutral low arousing pictures mostly in occipital and parietal cortical networks [8,18,19] indicating the allocation of attentional resources to stimuli according to their affective significance.

However, as these studies have examined the difference between the activation either in terms of an average over the whole steady-state stimulation interval or by reporting the time point of maximum difference [18] it is still unclear which epochs in the interval account for the effect found in the average.

In order to shed light on the temporal and spatial relation of cortical activation, we applied a complex demodulation procedure to minimum norm estimations of cortical activation. The complex demodulation waveform creates an envelope around the baseline-to-peak amplitude of the modulating steady-state signal with high temporal accuracy [17] and thus allows for accurate analysis of the temporal characteristics of the ongoing oscillations. We used Magnetoencephalography (MEG) to measure steady-state visual evoked fields (ssVEFs) during the four second interval of presentation of standardized affective pictures. Pleasant, unpleasant and neutral pictures from the International Affective Picture System (IAPS) [20] were chosen as stimulus material to allow for determining valence-related differences in stimulus processing. Minimum norm estimates (MNE) [21] were used to estimate the cortical sources of emotion-modulated ssVEFs.

We hypothesized that the peak of activity is not fixed and restricted to primary visual areas but rather shifts as higher order attentional processes come into play. In this way, we tried to analyze the time course of visual processing and attention in the time and space domain over the four second presentation interval. The sources of the signal contribution are expected to be located in posterior cortical areas, though not spatially fixed to the primary visual cortex. This represents the alterations of visual processing by emotional content and the interaction with higher order visual cortical areas.

## Methods

### Subjects

Seventeen right-handed participants (10 female) with mean age (29.9 +/- 6.4 years) and with normal or corrected-to-normal visual acuity gave informed consent to participate in the study. Subjects reported no history of photic epilepsy, had not experienced recent critical life events and had no history of psychotherapy or current psychopathology. The participants received 30 Euro for participation. The ethics committee of the University of Konstanz approved the procedures.

### Stimuli

Seventy-five colored pictures were chosen on the basis of their normative ratings from the International Affective Picture System [22]. Of these, 25 pictures presented unpleasant events (e.g. mutilations, assaults, etc.), 25 showed pleasant events (e.g. sports, erotic couples, children, etc.) and 25 showed neutral events (e.g. neutral faces, household objects, etc.). The three categories differed significantly from each other in IAPS normative valence ratings (pleasant: 7.4, neutral: 4.9, unpleasant: 2.4).

Normative arousal ratings did not differ for pleasant and unpleasant contents, but mean arousal levels for both emotional categories were significantly higher than for neutral contents (pleasant: 5.6, neutral: 2.9, unpleasant: 5.8.) Brightness, contrast and color spectra of the stimuli were matched across picture categories.

Pictures were presented with a video projector (JVC™, DLA-G11E) with a refresh rate of 100 Hz on a white plastic screen attached to the ceiling of the room. Pictures subtended a visual angle of 10° horizontally and 8° vertically to either side from the center of the screen. In each trial, one picture was presented in a flickering mode of 10 Hz for four seconds, resulting in 40 on/off cycles (same picture shown and not shown) of 50 milliseconds each. The inter-trial interval varied randomly between 6 to 8 seconds. In the inter-trial interval a grey screen with a fixation cross was presented to aid participants in maintaining gaze on the center of the screen.

### Procedure

Upon arriving at the laboratory, participants were familiarized with the MEG chamber and an informed consent form was signed. Handedness was determined using the Edinburgh Inventory [23]. For artifact control, four electrodes for the electro-oculogram (EOG) were attached; two near the left and right outer canthus and two above and below the right eye. Two electrodes attached at the left and right lower forearm recorded the electrocardiogram, which was monitored during the recording. As the aim of the current study was to introduce a rather new method of analyzing the spatial and temporal course of visual evoked brain activation, the presentation of ECG data would have gone beyond the scope of the paper. Results from the ECG recordings and correlations with several psychological and neural markers will be reported in an additional article. Subjects were then seated in a magnetically shielded chamber and their head shapes were digitized with a Polhemus 3Space Fasttrack (Polhemus, Colchester, VT, USA). Five index points (left and right periauricular points, nasion, pseudo-Cz and pseudo-inion point at the forehead) were determined to calculate the relative head position within the MEG helmet for source analysis. Finally, subjects were placed under the MEG sensors and instructed to avoid eye movement during picture presentation. A video camera monitored subjects' behavior and assured compliance throughout the experiment.

Then, the screen was positioned in front of the subjects and the presentation of 75 flickering (10 Hz) stimuli started. After MEG recordings, subjects rated each of the 75 affective pictures regarding emotional valence and arousal using the Self-Assessment Manikin self-report scale [22].

### MEG recording

Magnetic brain activity was recorded using a 148 channel whole-head system (Magnes™ 2500 WH, 4D Neuroimage, San Diego, USA). Vertical eye movements and blinks were recorded using Ag/AgCl-electrodes attached above and below the right eye (vertical electrooculogram). Lateral eye movements were recorded using two of the aforementioned electrodes at the outer canthi (horizontal electrooculogram). Electrocardiogram was recorded with two of the same electrodes on the left and right lower forearm. The ECG and EOG data were amplified using Synamps (Neuroscan™) Amplifiers. The MEG, ECG and EOG data was recorded with a sample rate of 678.17 Hz and filtered online with a band pass filter between 0.1 Hz and 200 Hz.

Procedures included in the MEG acquisition software package (Whole Head System software, version 1.2.5; 4D Neuroimaging) corrected global external noise and cardiac artifacts. Eye artifacts were corrected using the algorithm implemented in BESA™ software [24]. Trials containing large blink or EMG artifacts or maximum amplitudes above 3.5 pT were discarded from further analysis. The MEG data were digitally band pass filtered between 1 Hz and 25 Hz (slopes: 6 and 24 dB/octave, respectively) before averaging for picture category over 5000 ms (500 ms pre-stimulus, 4000 ms stimulus presentation and 500 ms post-stimulus).

### Data Analysis

The data analysis was carried out in two steps: First, the mean amplitude of the 10 Hz component was assessed using a moving window approach. Second, the time course of the modulation of the 10 Hz component over the four second interval of picture presentation was estimated using a complex demodulation technique.

### Moving Average

For each category average, the 10 Hz Fourier component was derived using a moving window averaging procedure [8]. To avoid contamination of results with the event related early activity, the initial 500 ms of the picture presentation interval were excluded. The resulting 500 – 4000 ms post stimulus part of each epoch was baseline-corrected using the 500 ms pre-stimulus interval. A 400-ms window containing four cycles of the 10 Hz flickering stimuli was shifted in steps of 100 ms (one cycle) across the epoch, and the magnetic field data within the shifting windows in the time domain were further averaged.

The resulting four cycles per category, subject and MEG channel were submitted to the fast Fourier-transformation (FFT) technique [25]. The real and the imaginary parts of the 10 Hz Fourier component were extracted for further analysis.

### Minimum Norm Estimation for the Moving Average Data

The real and imaginary parts of the 10 Hz Fourier component per condition resulting from the procedure mentioned above were submitted to minimum norm source estimation and subsequently recombined by taking the square root of the sum of the two squared dipole orientations. Cortical sources were estimated using the L2 minimum norm estimate (MNE), following the approach suggested by Hauk et al. [26] using EMEGS [27]. The L2-minimum-norm estimate enables enhanced resolution of brain activations generating the magnetic field without a priori assumptions regarding the location and number of current sources [21]. Calculation of the L2 minimum norm was based on a one-shell spherical head model with 2 (azimuth and polar direction) by 197 evenly distributed dipolar sources. This calculation was based on information on the center of a fitted sphere to the digitized head shape and the positions of the MEG sensors relative to the head. A spherical shell (1 shell, 6 cm, 197 dipoles) with evenly distributed dipole locations then served as source space. This shell was chosen as a compromise between depth sensitivity and spatial resolution [26]. The regularization parameter λ was .02 and thus identical across all subjects and conditions. After computing the minimum norm estimation for the real and imaginary parts of the 10 Hz Fourier component, both values were combined by using the square root of the sum of squares of the two Fourier parts as an estimate of absolute power [28].

### Minimum Norm Estimation for the assessment of time course

In order to assess the time course of the steady-state activation, a complex demodulation procedure was applied to minimum norm estimation data. Therefore, in a first step, the minimum norm estimation was computed for the four second interval of picture presentation. Here, we applied the same L2-minimum-norm technique as mentioned above, with the difference, that a minimum norm estimation was computed for every sample point in the raw data (3391 in total). All other parameters were kept equal. In a second step, the time course of the relevant 10 Hz component was extracted using a complex demodulation procedure. The detailed procedure is described below.

### Time course assessment

The time course of the amplitude of the 10 Hz steady-state component was computed separately for each dipole using the complex demodulation procedure. This procedure allows reliable extraction of the alterations of the amplitude of an ongoing waveform [29,30]. The complex demodulation mathematically extracts a modulating signal from a carrier signal by multiplying the raw data with a sine and cosine of the desired frequency and subsequent band pass filtration. The complex demodulation is computed as follows:



These two functions are applied to the averaged MEG raw data (MEG(t)). The frequency F in this case represents the driving frequency (in this case 10 Hz). Then, a 2 Hz Butterworth-filter is applied. The amplitude A(t) of the modulating signal is then described using the formula:



Finally, a baseline correction is applied in the same step using the 500 ms pre-stimulus baseline interval.

### Statistical Analysis

As a result of the aforementioned procedures, we obtained two different outcomes: First, we received the mean amplitude of the 10 Hz Fourier coefficient for every dipole as a measure for the averaged activation of the steady-state signal. Second, the complex demodulation procedure was used to derive the amplitude of the 10 Hz signal (component) for every sample point within the four second data interval as a measure of the time course of the activation for each of the 197 projected dipoles. The main goal of the statistical analysis of the MNE data was to show differences between the activation towards the different picture categories. For this purpose, we calculated pair-wise comparisons of the source activities for the three conditions. Condition-dependent activity was reflected by the contrast between activation towards affective (pleasant and unpleasant) and neutral pictures. To test for significant differences between the dipole activation of the three picture categories, we computed permutation tests. This procedure is qualified to cope with the high number of comparisons on dipole level without predetermined regions of interests [31].

Although no formal correction for multiple comparisons (Type 1 error) was made, only temporal and spatial regions comprising several sample points or dipoles respectively were interpreted, thus controlling for by-chance differences.

The advantage of the permutation test is that it does not require any a priori assumption about the distribution of the data, as it generates all possible permutations of the data to represent the data distribution. For each pair-wise condition comparison, we determined cut-off values for significant differences of the condition contrast at single dipole location based on 1000 (moving average) and 500 (time course) draws, respectively. For each draw, the individual condition contrast maps were randomly exchanged to generate data for a random condition composition. As we aimed at two-tailed tests, the maximum as well as the minimum of the differences at all dipole locations obtained from each draw entered the distributions of 1000 (500 respectively) maximum and minimum difference values. The upper and the lower critical values were determined as the 2.5% lowest and highest value in this distribution. Taken together, these two 2.5% tails represent critical limits of the 95% significance level (p < 0.05). In order to assess the time course, this was done successively for each sample point of the four second ssVEF interval. Difference values with permutation p < 0.05 were plotted onto a standardized brain. In order to accomplish this, the upper and lower critical difference values were subtracted from the original difference (unpleasant vs. neutral and pleasant vs. neutral) values. Thus, values greater than 0 for the upper critical value and less than 0 for the lower critical value represent the regions and epochs containing significant differences. This yielded maps of significant differences for each sample point between the two affective and the neutral conditions representing the main effect for condition under the null hypothesis that no difference between the conditions exists.

Müller et al.[17] noted that the greatest steady-state evoked potentials are found at occipital scalp sites. The aim of the present study was to evaluate the course of the activation over time. This includes the assumption, that the activation elicited by the steady-state stimulus is not spatially fixed.

## Results

As mentioned above, the following results were obtained using a permutation test yielding significant differences above the 95%-level. Unless stated differently, all results relate to this level of significance. Due to this, no differentiation between levels of significance is being made within the plots. With respect to the mean amplitude estimates, the results of the permutation tests were confirmed using repeated measures ANOVA. The results of the time course analysis were confirmed by point wise t-tests. Where appropriate, the degrees of freedom were corrected in all ANOVA analyses using the Greenhouse and Geisser [32] procedure to account for possible violations of the sphericity assumption. Bonferroni post-hoc comparisons were used to investigate significant interaction effects.

### Mean Amplitude

**Figure 1:**
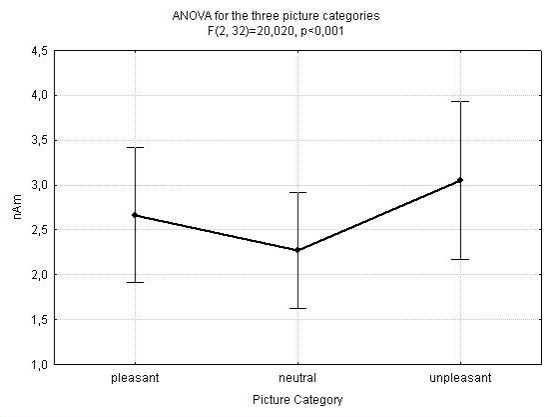
**Comparison Unpleasant vs. Neutral vs. Pleasant Stimuli**. Mean condition dependent activation (in nAm) and SE towards high arousing unpleasant, neutral and pleasant slides at the occipital ROI.

The estimation of the mean amplitude differences between the three affective categories revealed significantly higher amplitudes towards arousing pictures in the occipital lobe. Also, the unpleasant pictures elicited higher activation compared to the pleasant images. As illustrated in figure 1, the repeated measures ANOVA with the factor condition (pleasant, neutral and unpleasant) confirmed these results (F (2, 32) = 20.02, p < 0.001, Bonferroni p < 0.05 for all comparisons).

**Figure 2:**
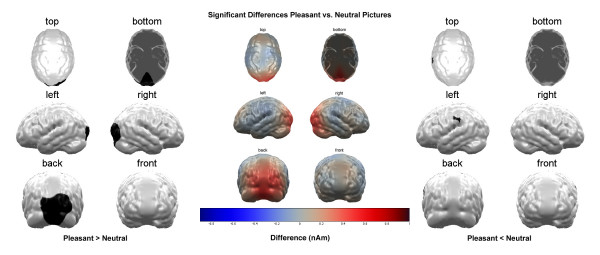
**Comparison Pleasant vs. Neutral Stimuli**. Brain maps showing significant condition differences with respect to cortical source activation towards pleasant and neutral picture content. Depicted are the significant differences in the average over the whole stimulation interval as calculated in the permutation analysis. The left panel shows the areas, where pleasant stimuli lead to higher activation, the right panel shows the areas where neutral stimuli lead to higher activation and the center panel shows the difference of activation that was fed to the permutation analysis.

**Figure 3:**
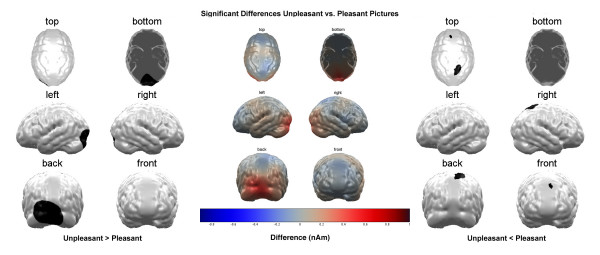
**Comparison Unpleasant vs. Pleasant Stimuli**. Brain maps showing significant condition differences with respect to cortical source activation towards unpleasant and pleasant picture content. Depicted are the significant differences in the average over the whole stimulation interval as calculated in the permutation analysis. The left panel shows the areas, where unpleasant stimuli lead to higher activation, the right panel shows the areas where pleasant stimuli lead to higher activation and the center panel shows the difference of activation that was fed to the permutation analysis.

**Figure 4:**
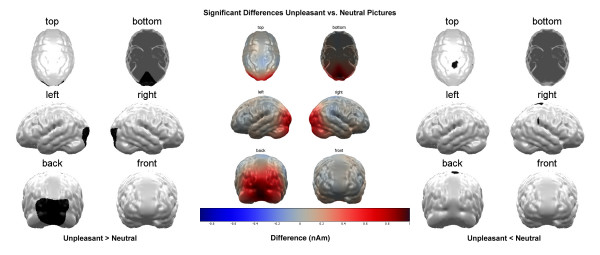
**Comparison Unpleasant vs. Neutral Stimuli**. Brain maps showing significant condition differences with respect to cortical source activation towards unpleasant and neutral picture content. Depicted are the significant differences in the average over the whole stimulation interval as calculated in the permutation analysis. The left panel shows the areas, where unpleasant stimuli lead to higher activation, the right panel shows the areas where neutral stimuli lead to higher activation and the center panel shows the difference of activation that was fed to the permutation analysis.

While the pleasant pictures caused a lower activation in the right parieto-temporal cortex compared to the neutral pictures (t (16) = -2.82, p < 0.01; see figure 2), activity in a parietal area was reduced for the unpleasant pictures compared to the pleasant (t (16) = -1.21, p = 0.23; see figure 3) as well as the neutral pictures (t (16) = -1.29, p = 0.2; see figure 4). The small t-values in these comparisons are due to the large inter-individual variance in these cortical areas.

### Time course

The results of the permutation tests identified significant differences between the affective categories and the neutral pictures. Two .mpeg video files illustrate the change of brain activity over the course of the four seconds of picture presentation [see additional file 1 for the unpleasant – neutral comparison and additional file 2 for the pleasant – neutral comparison]. These videos exemplify that over time the significant differentiation between the affective categories shifts in space and magnitude. The time course of three regions of interest (occipital, right temporal and parietal) was subsequently analyzed using t-tests to confirm the results obtained by the permutation test. As the results of the comparison of activation towards unpleasant and neutral pictures gave rise to stronger effects, the results of the comparison of pleasant and neutral stimuli will not be discussed explicitly.

**Figure 5:**
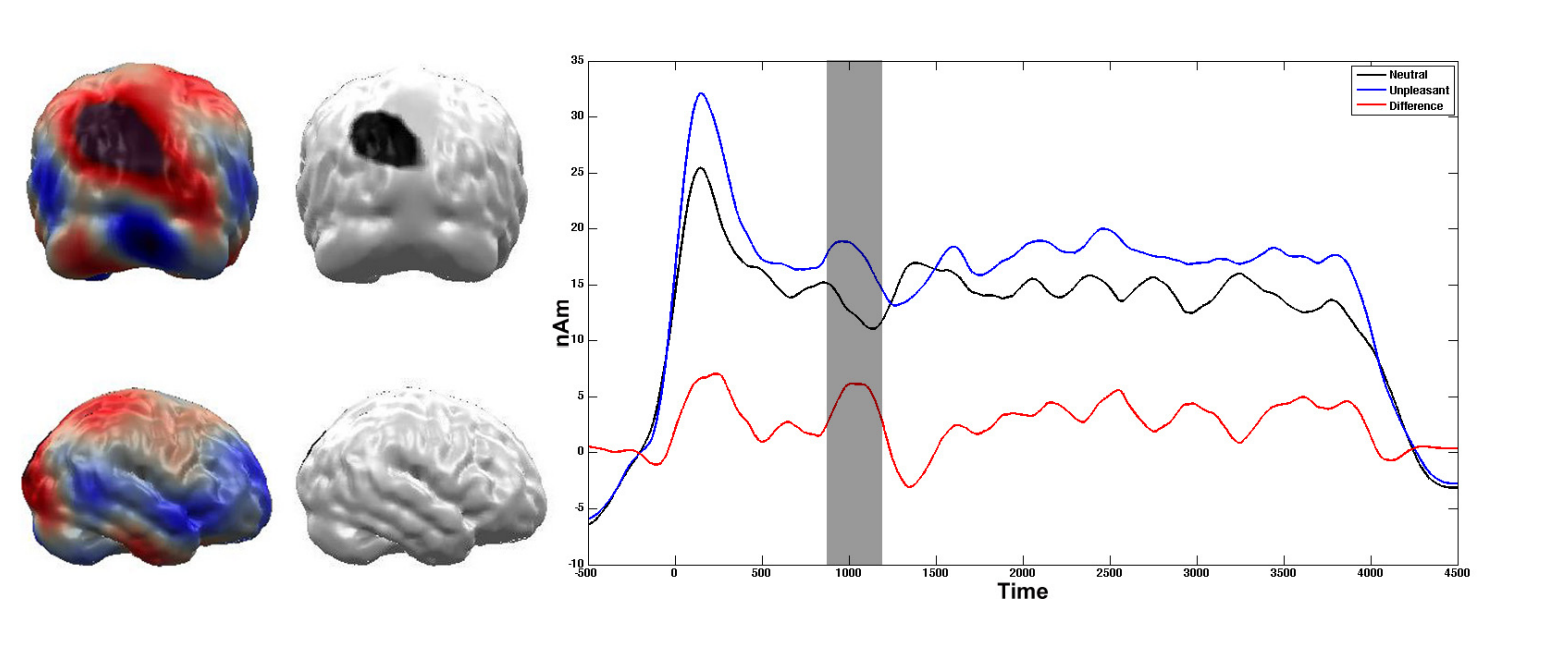
**Comparison between Unpleasant vs. Neutral Stimuli in the occipital ROI**. Time course of condition dependent activation (in nAm) towards high arousing unpleasant and neutral slides at the occipital ROI. Grey areas in the time course plot mark intervals of significant t-comparisons. Brain plots illustrate the ROI (right panel) as well as the mean difference of activation of the marked interval (left panel).

**Figure 6:**
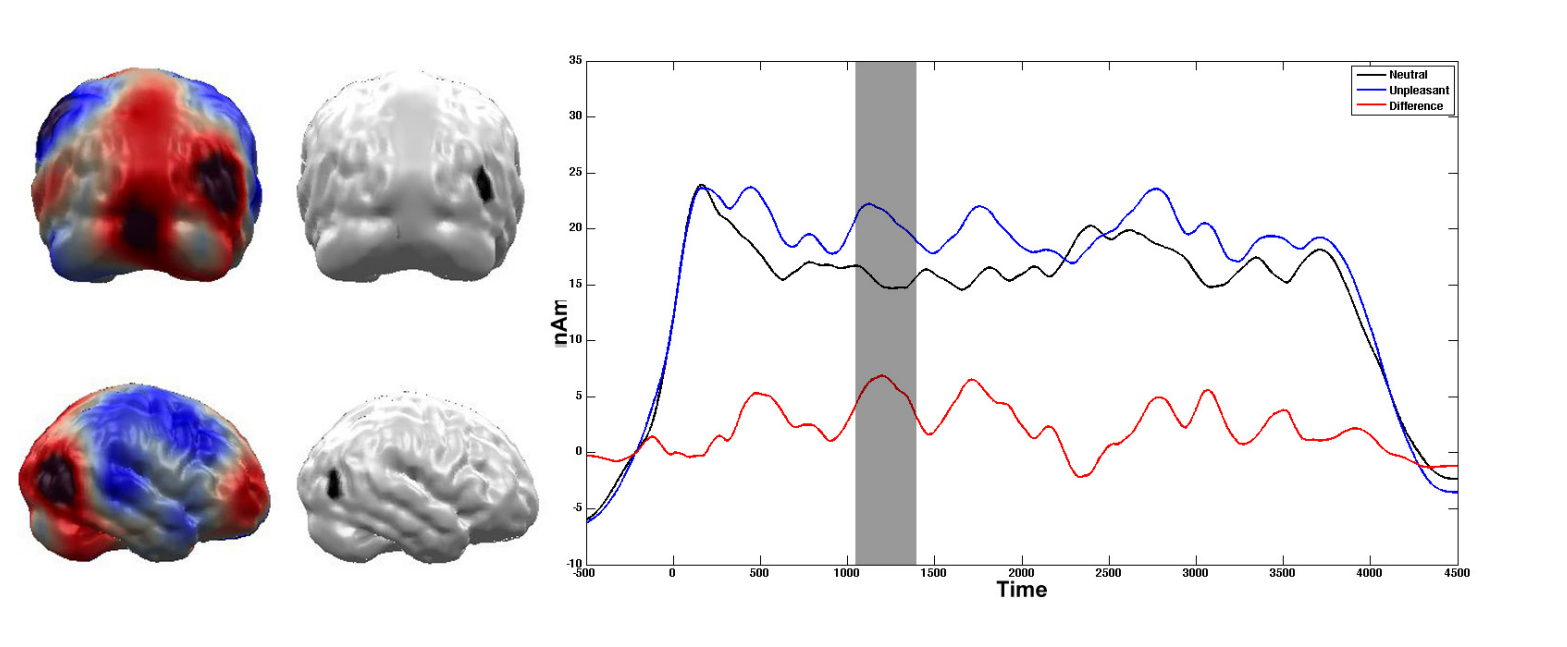
**Comparison between Unpleasant vs. Neutral Stimuli in the right temporal ROI**. Time course of condition dependent activation (in nAm) towards high arousing unpleasant and neutral slides at the right temporal ROI. Grey areas in the time course plot mark intervals of significant t-comparisons. Brain plots illustrate the ROI (right panel) as well as the mean difference of activation of the marked interval (left panel).

**Figure 7:**
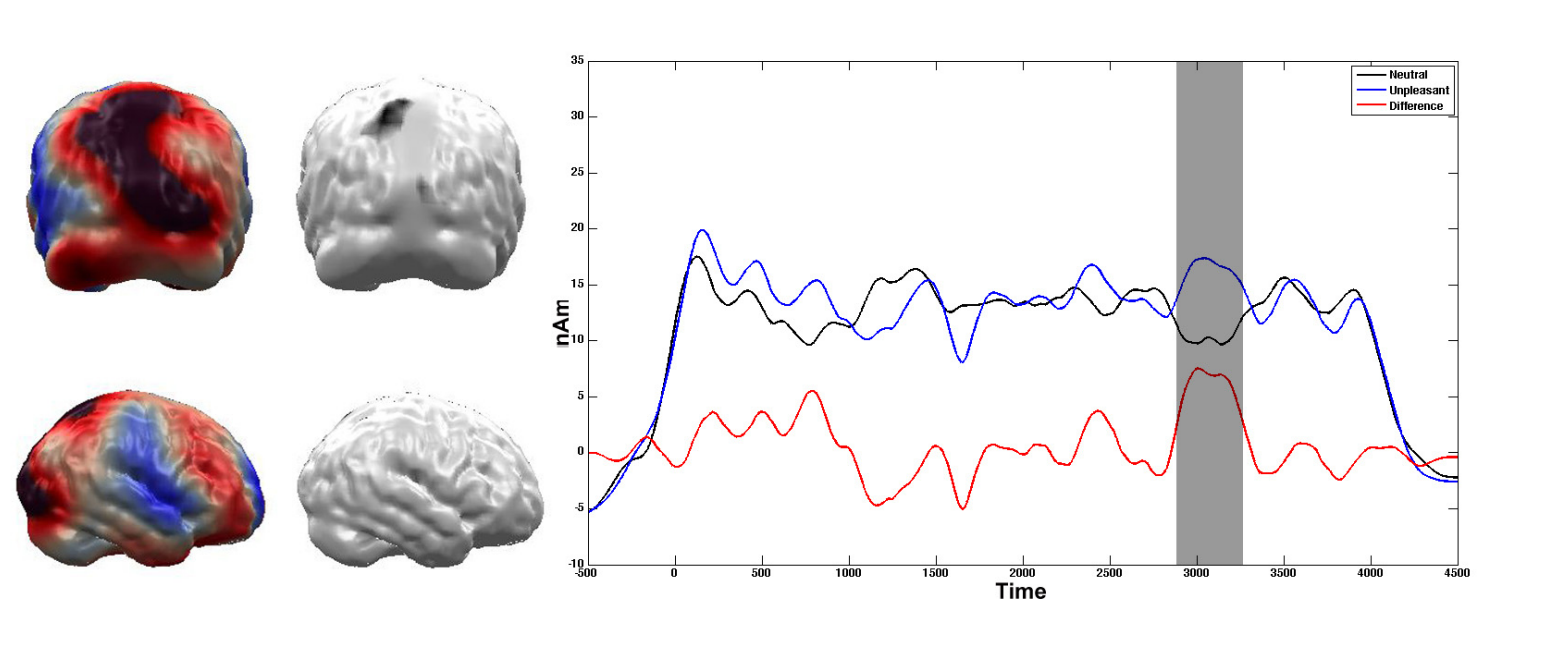
**Comparison between Unpleasant vs. Neutral Stimuli in the parietal ROI**. Time course of condition dependent activation (in nAm) towards high arousing unpleasant and neutral slides at the parietal ROI. Grey areas in the time course plot mark intervals of significant t-comparisons. Brain plots illustrate the ROI (right panel) as well as the mean difference of activation of the marked interval (left panel).

Significant differences between the unpleasant and the neutral pictures were obtained from the very beginning of the stimulation. As these early components are subject to a tapering procedure in the complex demodulation procedure, the first 500 ms post stimulus have to be interpreted with caution and are thus not discussed here. The first differentiation between the affective categories following the initial ERFs was found between 938 ms and 1174 ms post stimulus (t (16) = 2.79, p < 0.05). This difference in brain activation within the first second was restricted to the primary visual cortex (see figure 5). The unpleasant pictures elicited higher amplitudes in the primary visual cortex compared to neutral pictures. Over the course of the four second picture presentation, this higher activity regarding affective pictures shifted towards extrastriate cortex in occipito-temporal and posterior parietal cortex areas (see figures 6 and 7). After the initial processing in the primary visual cortex, the differentiation was found between 1140 ms and 1320 ms post stimulus in a temporal region (t (16) = 2.97, p < 0.01, see Figure 6). This difference between unpleasant and neutral stimuli was found in a parietal region between 2950 ms and 3235 ms post stimulus (t (16) = 3.83, p < 0.01, see Figure 7).

The pleasant pictures also led to a higher activation in occipital brain regions, although this differentiation was not as pronounced as with the unpleasant pictures. The spatial allocation of activation in the occipital cortical regions was roughly equivalent in response to the pleasant and unpleasant pictures. The initial differentiation was again found in the primary visual cortex and subsequently shifted towards the extrastriate cortex. Pleasant and unpleasant pictures consistently lead to higher amplitudes than neutral images.

## Discussion

The present study aimed at establishing an accurate and parsimonious paradigm to analyze physiological data over the time course. The paradigm has been applied to the replication and extension of previous findings from studies regarding processing of affective pictures. Consistent with previous research, we found higher activation over occipital areas towards high-arousing pictures in healthy subjects. Both, studies examining ssVEF – as well as ssVEP-changes as a function of emotional arousal have shown greater amplitudes in regions involving the occipital cortices and temporo-parietal cortices [8,18,19]. These findings are typically discussed in the framework of selective attention suggesting that more attentional resources are allocated to external stimuli according to their affective significance to enhance sensory processing of relevant information [1]. Hillyard et al. [33] proposed, that attentional processes may be subject to a mechanism of gain control. The authors described a mechanism, which gives input from an attended locations an improved signal-to-noise ratio so that more information can be extracted from relevant proportions of the visual field. Keil et al. [9] suggested, that the cortical networks for sensory processing might be subject to a gain mechanism according to the motivational relevance as well. That means, that not only the attentional relevance but also the motivational relevance (e.g. the fast reaction to potentially threatening stimuli) of a stimulus amplifies the sensory processing of a stimulus. Or to state it differently, motivationally relevant stimuli naturally and perhaps automatically arouse and direct attentional resources [1].

The results of the moving average procedure clearly replicate these aforementioned findings as well as those by Moratti et al.[19]. Emotional stimuli elicit greater ssVEF amplitudes compared to neutral stimuli. The results of the time course evaluation also replicate the findings from Müller et al.[17], that the greatest differentiation can be recorded at occipital sites.

In addition to these results, we showed that the difference between the activation produced by the affective and neutral pictures fluctuates over time and that the location of the differentiation changes over time. While the results from the moving average procedure point to the primary visual cortex in the occipital region as the area of greatest differentiation between the picture categories, the results of the time course analysis show the additional involvement of extrastriate parietal and temporal brain regions. This is especially important when choosing regions of interest or intervals of interest over which an average is being computed.

With our analysis we could show that affective modulation of cortical activity is not spatially fixed, as can be seen in the different brain maps over the time course. Although the peak of activity can initially be seen in the primary visual cortex within the central occipital region, the location of the peak shifts over time towards secondary and associate visual cortices. This could explain why Müller et al [17] could not find a significant difference between the activation following affective and neutral images after 1500 ms post stimulus based on the measurement of a single electrode. Assuming a fixed region of interest for the steady-state data does not allow revealing topographically changing activity that was found by our analysis. However, the several technical restrictions limit the power of the procedure. As mentioned above, tapers are applied in the complex demodulation procedure. Due to these graduations at the beginning and the end of the resulting waveform, it is not possible to correctly estimate the activity measured in the first and last 500 ms of the stimulus interval. Conventional ERF analysis procedures are better suited to address questions concerning these early potentials, while the complex demodulation procedure is aimed at investigating long-term modulations.

Also, the temporal resolution of the complex demodulation waveform is diffused due to the filtering. Hence, the exact timing of spatial changes in activation is somewhat distorted while the progression of activation is correct. Still, this procedure is able to assess these temporal and spatial changes more accurately than fMRI. While the combination of fMRI and VEP-measurements promise to measure both, fast event-related processing and related hemodynamic changes, it is possible, that two different processes occur. When measuring steady-state evoked fields on the contrary, it is possible to examine the spatial and temporal features of cortical information processing with one analytical method.

## Conclusion

Studies using fMRI have identified a variety of cortical areas involved in the processing of emotional information. ERP analyses have suggested a sequential pattern of processing that occur in very brief time periods. Here, we have extended these findings by providing detailed information about the spatial changes over time as well as the temporal characteristics of information processing. Within the first four seconds, the affective modulation of cortical activity is not spatially fixed, but changes locations in occipital, parietal and temporal regions. In this study, we showed that the peaks of affective modulation of cortical activation are unstable already within the first four seconds of stimulus processing. This finding indicates that current models of brain activity based on imaging techniques with a low temporal resolution might be too simplified. The application of a variety of techniques allowing different levels of spatial and temporal resolution is necessary to explore the implications of the temporal variation of cortical activity. The temporal analysis of the ssVEP signal can be one promising tool to obtain more realistic models of brain activity.

## Authors' contributions

JK developed the analytical approach, carried out the data pre processing, source estimation, statistical analysis and drafted the manuscript. HA developed the analytical approach, carried out the MEG recordings and clinical interviews with the participants and helped to draft the manuscript. CC designed the experiment and participated in the statistical analyses and helped to draft the manuscript. FN conceived the design of the study and statistical analyses and coordinated and helped to draft the manuscript. All authors read and approved the final manuscript.

## Appendix

The numbers of IAPS pictures were as follows:

Pleasant: 2190, 2214, 2215, 2383, 2440, 2480, 2516, 2840, 2850, 5130, 5510, 5740, 7035, 7175, 7217, 7491, 7500, 7590, 7595, 7700, 8190, 5830, 5660, 4607, 2209.

Neutral: 1722, 2030, 2058, 2165, 2216, 2311, 2340, 2345, 2352, 4599, 4608, 4641, 4653, 4660, 5260, 5700, 8185, 8200, 8380, 8496, 7490, 7130, 5390, 2570, 2410.

Unpleasant: 2120, 2900, 3181, 3301, 6190, 6212, 6250, 6312, 6540, 6560, 6831, 6838, 9040, 9181, 9400, 9405, 9415, 9421, 9433, 9911, 6821, 3550, 3530, 2800, 2053.

## Supplementary Material

Additional file 1 Video of the time course of difference of activation between unpleasant and neutral pictures over the 4000 ms stimulation interval. Can be viewed with any standard video player. Click here for file

Additional file 2 Video of the time course of difference of activation between pleasant and neutral pictures over the 4000 ms stimulation interval. Can be viewed with any standard video player. Click here for file
